# T1 mapping, ECV and ICV before and after aortic valve replacement

**DOI:** 10.1186/1532-429X-17-S1-P342

**Published:** 2015-02-03

**Authors:** Emmanuelle Vermes, Nicolas Cazeneuve, Olivier Genee, Anne Delhommais, Laurent Brunereau, Daniel Alison, Julien Pucheux

**Affiliations:** 1 Cardiac Imaging, CHU Trousseau, Tours, France

## Background

the aim of the study was to assess T1 mapping using T1 values, extracellular volume (ECV) and intra cellular volume (ICV) with cardiovascular magnetic resonance (CMR) in patients with surgical aortic stenosis before and after surgery.

## Methods

A prospective CMR T1 mapping study of 37 patients with severe AS before and six months after aortic valve replacement (AVR) was conducted. CMR at 1.5 T, including T1 mapping using a modified Look-Locker inversion recovery sequence (before and 15 minutes after the administration of 0.2 mmol/kg of Gadoteric acid), was carried out. Global T1 values, ECV, ICV have been measured before and six months after surgery.

## Results

After AVR, post contrast T1 values were significantly lower compared to before surgery (443 ± 18 ms versus 460 ± 19 ms; p=0,027). ECV was significantly higher after surgery (27,9 ± 4,7 % versus 25,1 ± 2,7 %; p = 0.0001). Reciprocally, ICV significantly decreased after surgery (74,9 % versus 72,1 % p = 0.0001). In parallel, myocardial mass (89.5 ± 27 g/m2 versus 74.5 ± 23 g/m2 ; p<0,001), end diastolic volume (74 ± 19 ml/m2 versus 65 ± 12 ml/m2 ; p<0,001) and septal thickness (15 ± 2.5 mm versus 14 ± 2.5 mm ; p<0,001) decreased significantly after surgery.

## Conclusions

Post contrast T1 values, ICV, myocardial mass and septal thickness significantly decreased after AVR, suggesting that LV hypertrophy regression is more cellular than interstitial fibrosis regression. Larger studies are needed to confirm these data.

## Funding

No funding.

